# Chemical modification of melphalan as a key to improving treatment of haematological malignancies

**DOI:** 10.1038/s41598-020-61436-x

**Published:** 2020-03-11

**Authors:** Arkadiusz Gajek, Anastazja Poczta, Małgorzata Łukawska, Violetta Cecuda- Adamczewska, Joanna Tobiasz, Agnieszka Marczak

**Affiliations:** 1 Department of Medical Biophysics, Institute of Biophysics, Faculty of Biology and Environmental Protection, University of Lodz, Pomorska 141/143, 90-236 Lodz, Poland; 2 ŁUKASIEWICZ Research Network-Institute of Biotechnology and Antibiotics, 5 Staroscinska St., 02-516 Warsaw, Poland

**Keywords:** Drug development, Cell death, Drug screening

## Abstract

Chemical modification of known, effective drugs is one method to improve chemotherapy. Thus, the object of this study was to generate melphalan derivatives with improved cytotoxic activity in human cancer cells (RPMI8226, HL60 and THP1). Several melphalan derivatives were synthesised, modified in their two important functional groups. Nine analogues were tested, including melphalan compounds modified: only at the amino group, by replacing the amine with an amidine group containing a morpholine ring (MOR-MEL) or with an amidino group and dipropyl chain (DIPR-MEL); only at the carboxyl group to form methyl and ethyl esters of melphalan (EM-MEL, EE-MEL); and in a similar manner at both functional groups (EM-MOR-MEL, EE-MOR-MEL, EM-DIPR-MEL, EE-DIPR-MEL). Melphalan derivatives were evaluated for cytotoxicity (resazurin viability assay), genotoxicity (comet assay) and the ability to induce apoptosis (terminal deoxynucleotidyl transferase dUTP nick end labelling, TUNEL, phosphatidylserine externalisation, chromatin condensation, activity of caspases 3/7, 8 and 9 and intracellular concentration of calcium ions) in comparison with the parent drug. Almost all derivatives, with the exception of MOR-MEL and DIPR-MEL, were found to be more toxic than melphalan in all cell lines evaluated. Treatment of cultures with the derivatives generated a significant higher level of DNA breaks compared to those treated with melphalan, especially after longer incubation times. In addition, all the melphalan derivatives demonstrated a high apoptosis-inducing ability in acute monocytic and promyelocytic leukemia cells. This study showed that the mechanism of action of the tested compounds differed depending on the cell line, and allowed the selection of the most active compounds for further, more detailed investigations.

## Introduction

Cancer is considered to be one of the most serious health problems. Multiple myeloma (MM) is malignant plasma cell disorder that is characterized by the presence of clonal plasma cell proliferation in bone marrow and over production of monoclonal paraprotein in the blood and/or urine^1,2^.

The nitrogen mustards, as alkylating agents, belong to the earliest effective antitumor drugs used in cancer chemotherapy^3^. This cytotoxic drug exerts its pharmacological activity by inducing interstrand links in the main DNA groove, crosslinking of two strands, and preventing DNA replication, resulting in cell death. At the molecular level, individual pairs of nitrogen generate a strained intermediate “aziridinium ion” that is very reactive to cell DNA, and binds to the N7 nitrogen on the DNA base guanine. This linkage represents the most toxic of all alkylation events^4,5^. The alkylating agent- melphalan (MEL) has been used in the treatment of several types of haematological malignancies and solid tumor^6^. Nowadays, clinical usage of melphalan is limited to multiple myeloma treatment^7^. An observation that has fundamentally changed the standard of MM treatment, was that melphalan used in large doses (100 mg/m^2^ and more) breaks myeloma resistance to low doses very effectively. Melphalan at these doses also destroys healthy hematopoietic cells, so such treatment requires prior collection of these cells from the patient (usually from peripheral blood), storage and transplantation after melphalan administration (autologous stem cell transplantation, ASCT). This therapy involves significant toxicity and can therefore be used only in younger patients, not affected by significant accompanying diseases. Thus, the possibility of using this treatment has become the basis for the division of patients into two main categories: patients eligible for high doses of melphalan and transplantation of their own hematopoietic cells and patients not eligible for such treatment. High-dose melphalan (range: 140–200 mg/m^2^) plus ASCT currently serves as the standard treatment approach for patients with newly diagnosed, transplant-eligible multiple myeloma^1,2,6^.

The occurrence of drug resistant, lack of selectivity and high toxicity are the primary limiting factor for the long-term success of this treatment^2,3,8^. Side effects are mainly restricted to the bone marrow, though at very high doses it also involves the gastrointestinal tract and bone marrow supression^9^. For these reasons, there is an urgent need to develop new antitumour drugs for a wide variety of tumours, especially those showing primary resistance to conventional therapy, or that have developed resistance after treatment with conventional cancer chemotherapies.

Although much effort has been made to find new melphalan derivatives that will overcome all the above-mentioned problems and increase uptake of the drug into cells, none of them has found a clinical application^4,10,11^. Our previous studies have indicated that oxazoline and formamidine modification of anthracyclines are promising way of enhancing a drug, compared to its native form. The conclusion from that study was this type of improvement is detrimental to cancer cells, making it a potential approach in devising cell therapy strategies^12–18^. Although belonging to a completely different group of anticancer compounds, we decided to verify the effectiveness of these modifications for the anti-cancer activity of melphalan, and to select the most active compounds for further, more detailed studies. Due to the presence of two important functional groups on the melphalan molecule, modifications were made at both sites, -COOH and -NH2, to assess their effect on the cytotoxic properties of the compound. The derivatives were also assayed to determine which modification was the best. Our research aims not only to increase the antitumor activity of melphalan but also to extend its spectrum of activity (promyelocytic leukemia and acute monocytic leukemia).

Chemical modifications included a carboxyl group, with synthesised compounds being methyl and ethyl esters (EM-MEL, EE-MEL). In addition, these derivatives were modified at their amino groups by replacing the amine group with an amidine group containing a morpholine ring (EM-MOR-MEL, EE-MOR-MEL), or with an amidine group and dipropyl chain (EM-DIPR-MEL, EE-DIPR-MEL). For a systematic analysis of structure-activity relationships, melphalan derivatives modified only at the amino group were also added to the study. These modifications were made in the same manner as described above; however, the compounds were not modified at the carboxyl group (MOR-MEL, DIPR-MEL). This group of analogues were used to assess the potential anticancer properties of the structural changes in comparison to the parent compound, melphalan.

## Results

### Cytotoxicity of melphalan and its derivatives in selected human cell line models

**Figure 1:**
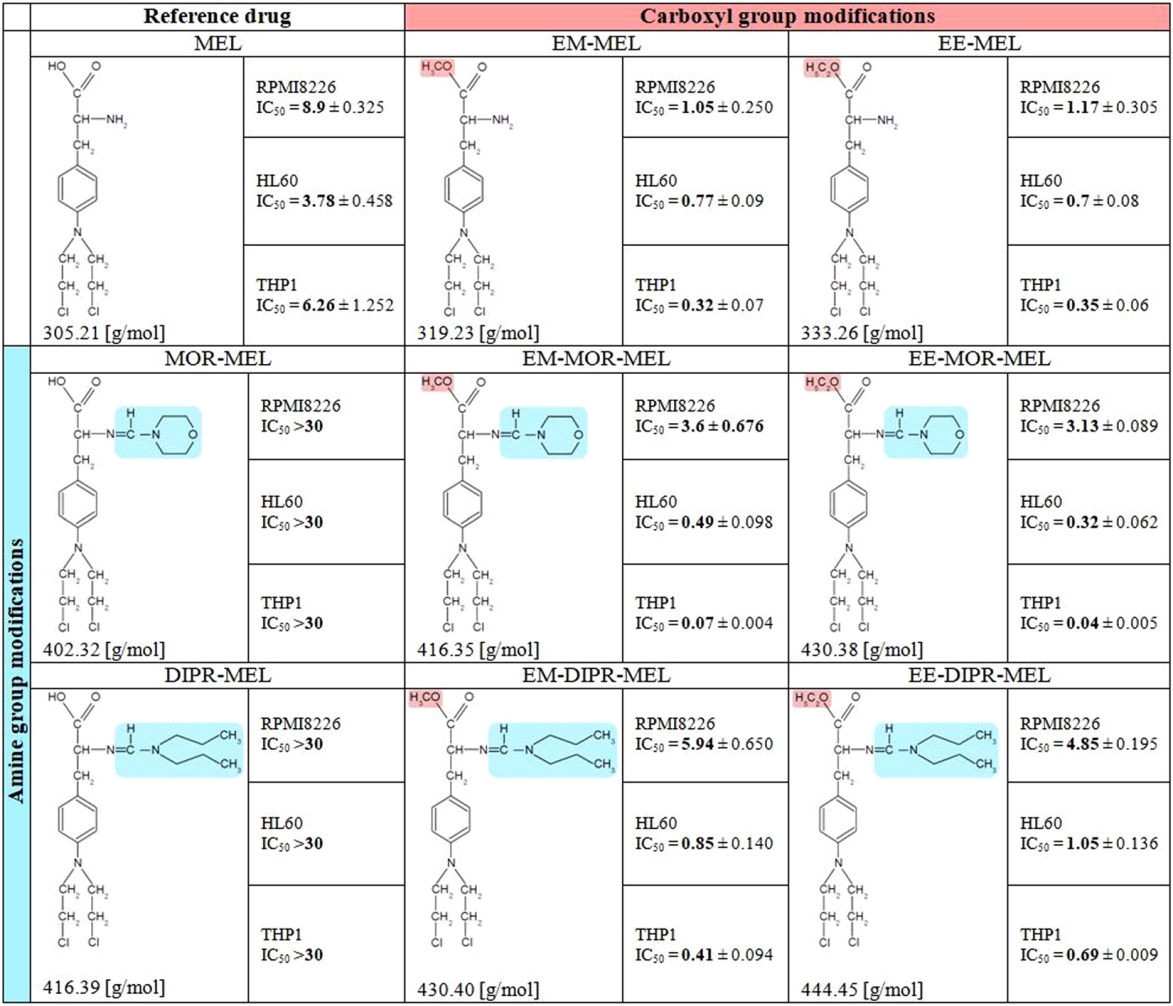
IC_50_ ± SD values [µM] of compounds in RPMI8226, HL60 and THP1 human cancer cell lines.

Sensitivities of myeloma cells (RPMI8226), acute promyelocytic leukemia cells (HL60) and acute monocytic leukemia cells (THP1) to melphalan and its derivatives were estimated from the drugs half maximal inhibitory (IC_50_) concentrations. Figure 1 shows the influence of the tested compounds on the viability of human cancer cells, as estimated by the resazurin viability assay.

The modifications of the melphalan molecule were designed to evaluate, the importance and desirability of modifications of melphalan at two functional groups of its molecule, with as few compounds as possible.

- *The cytotoxicity of the parent drug, melphalan* *(****MEL****)*
  The individual cell lines exhibited notably different sensitivities to the reference drug. The IC_50_ values for melphalan in RPMI8226, THP1 and HL60 cells were 8.9 µM, 6.26 µM and 3.78 µM, respectively.
- *The cytotoxicity of melphalan compounds modified only at the amino group by replacing the amine group with an amidine group containing a morpholine ring or an amidino group with a dipropyl chain* *(****MOR-MEL, DIPR-MEL****)*
  Chemical modifications only at the amine group abolish the cytotoxic activity of melphalan against all the investigated cancer cell lines. MOR-MEL and DIPR-MEL derivatives did not differentially influence cell viability to any measurable extent during the incubation period.
- *The cytotoxicity of melphalan compounds modified only at carboxyl group: methyl and ethyl esters of melphalan* *(****EE-MEL*** *and* ***EM-MEL****)*
  By contrast, modifications only at the carboxyl group had the opposite effect, with EM-MEL and EE-MEL derivatives showing the highest activity of any of the compounds against myeloma cancer cells. In general, melphalan esters were considerably more cytotoxic, having approximately an 8-fold lower IC_50_ concentration (1.05 µM for EM-MEL and 1.17 µM for EE-MEL) than the unmodified drug (8.9 µM) in RPMI8226 cell line, 5.5-fold lower IC_50_ concentration (0.77 µM for EM-MEL and 0.7 µM for EE-MEL) in HL60 cell line and 20-fold lower IC_50_ concentration (0.32 µM for EM-MEL and 0.35 µM for EE-MEL) in THP1 cell in comparison to MEL (3.78 µM-HL60 and 6.26 µM-THP1).
- *The cytotoxicity of melphalan compounds modified at both the amino and carboxyl functional groups* *(****EE-MOR-MEL, EM-MOR-MEL*** *and* ***EE-DIPR-MEL, EM-DIPR-MEL****)*

Cytotoxicity of EE-MOR-MEL, EM-MOR-MEL, EE-DIPR-MEL and EM-DIPR-MEL to RPMI8226 cancer cells were considerably lower than for melphalan compounds modified only at the carboxyl group. However, IC_50_ concentrations obtained for this group were still lower than that observed for MEL. EE-MOR-MEL showed the highest activity (IC_50_ = 3.13 µM), more than 2.8-fold that of melphalan. EM-MOR-MEL showed the second highest activity (IC_50_ = 3.6 µM) in this group. EE-DIPR-MEL and EM-DIPR-MEL had IC_50_ values of 5.94 µM and 4.85 µM, respectively, in both cases showing greater activity than MEL (IC_50_ = 8.9 µM).

By contrast, in leukemia cells, both investigated cell lines were significantly more sensitive to EE-MOR-MEL and EM-MOR-MEL than myeloma cancer cells. IC_50_ concentrations obtained for the most active compound (EE-MOR-MEL) were around 12- (HL60, IC_50_ = 0.32 µM) and 160- (THP1, IC_50_ = 0.04 µM) fold lower than that for the unmodified drug (MEL). EE-DIPR-MEL (IC_50_ = 1.05 µM in HL60, IC_50_ = 0.69 µM in THP1), EM-DIPR-MEL (IC_50_ = 0.85 µM in HL60, IC_50_ = 0.41 µM in THP1), though having lower cytotoxity, showed similar changes in IC_50_ values to the methyl and ethyl esters (EE-MOR-MEL, EM-MOR-MEL).

Taken together, these results indicate that the ester group was essential for the compounds high cytotoxicity. Additional substituents at the amino group, whether with an amidine group containing a morpholine ring or an amidino group with a dipropyl chain, decreased (RPMI8226 cells) or enhanced (THP1 and HL60 cells) the activity. Due to the observed toxic effects, measurements of phosphatidylserine externalisation, chromatin condensation, labelling of Br-dUTP to 3′OH ends of single- and double-stranded DNA fragments and comet assay experiments were performed for all compounds at concentrations of 0.3 μM for THP1 cells, 0.7 μM for HL60 cells and 3 µM for RPMI8226 cells.

We performed study to evaluate the cytotoxic effect of selected melphalan derivatives (EE-MEL, EM-MEL and EM-MOR-MEL) on normal peripheral blood-derived mononuclear cells (PBMC). Reduced cytotoxicity of the tested derivatives in PBMC and increased cytotoxic effect in tumor cell lines were observed. The IC_50_ values for esters of melphalan in PMBC (EE-MEL - IC_50_ = 2.20 ± 0.25 μM; EM – MEL - IC_50_ = 2.39 ± 1.08 μM) was around 4.5-fold higher than leukemia and 2-fold higher for multiple myeloma cells. The most beneficial effect (the biggest difference between normal and cancer cells) was noted for EM-MOR-MEL. The IC_50_ value was around 90-fold higher in PBMC (IC_50_ = 6.41 ± 0.85 μM) in comparison to THP1, 15-fold in HL60 and 2-fold in RPMI8226 cells.

### Evaluation of impact on parameters directly related to apoptotic processes - measurement of phosphatidylserine externalisation and chromatin condensation

**Figure 2:**
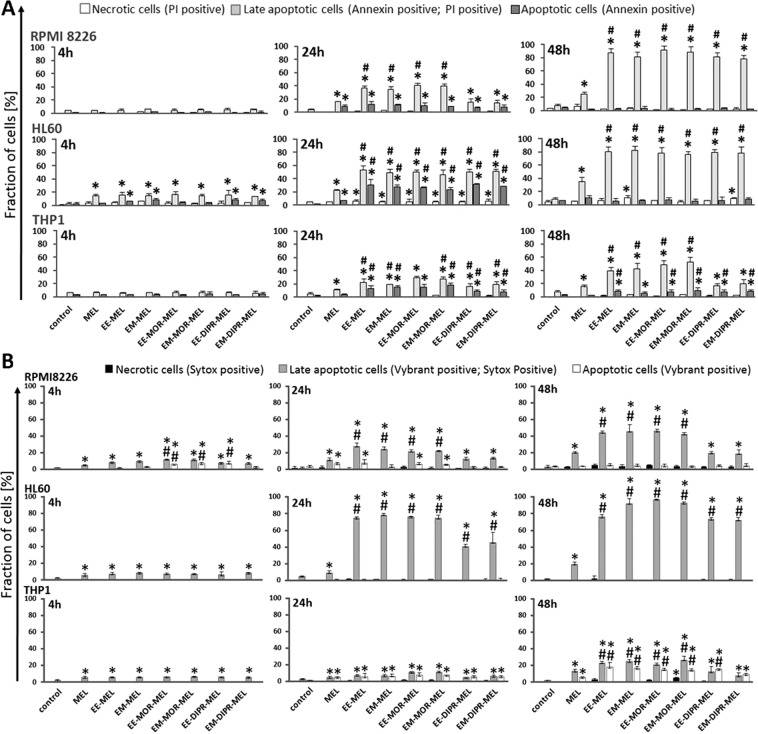
Influence of melphalan and its derivatives on the induction of apoptosis in RPMI8226, HL60 and THP1 cancer cell lines, as estimated by annexin V/propidium iodide (**A**) and Vybrant® DyeCycle™ Violet/SYTOX^®^ AADvanced^™^ (**B**) assays. Quantitative results of the compounds effect on the level of necrotic, and early and late apoptotic cells are represent by the mean ± standard deviation (SD) for three independent experiments.

**Figure 3:**
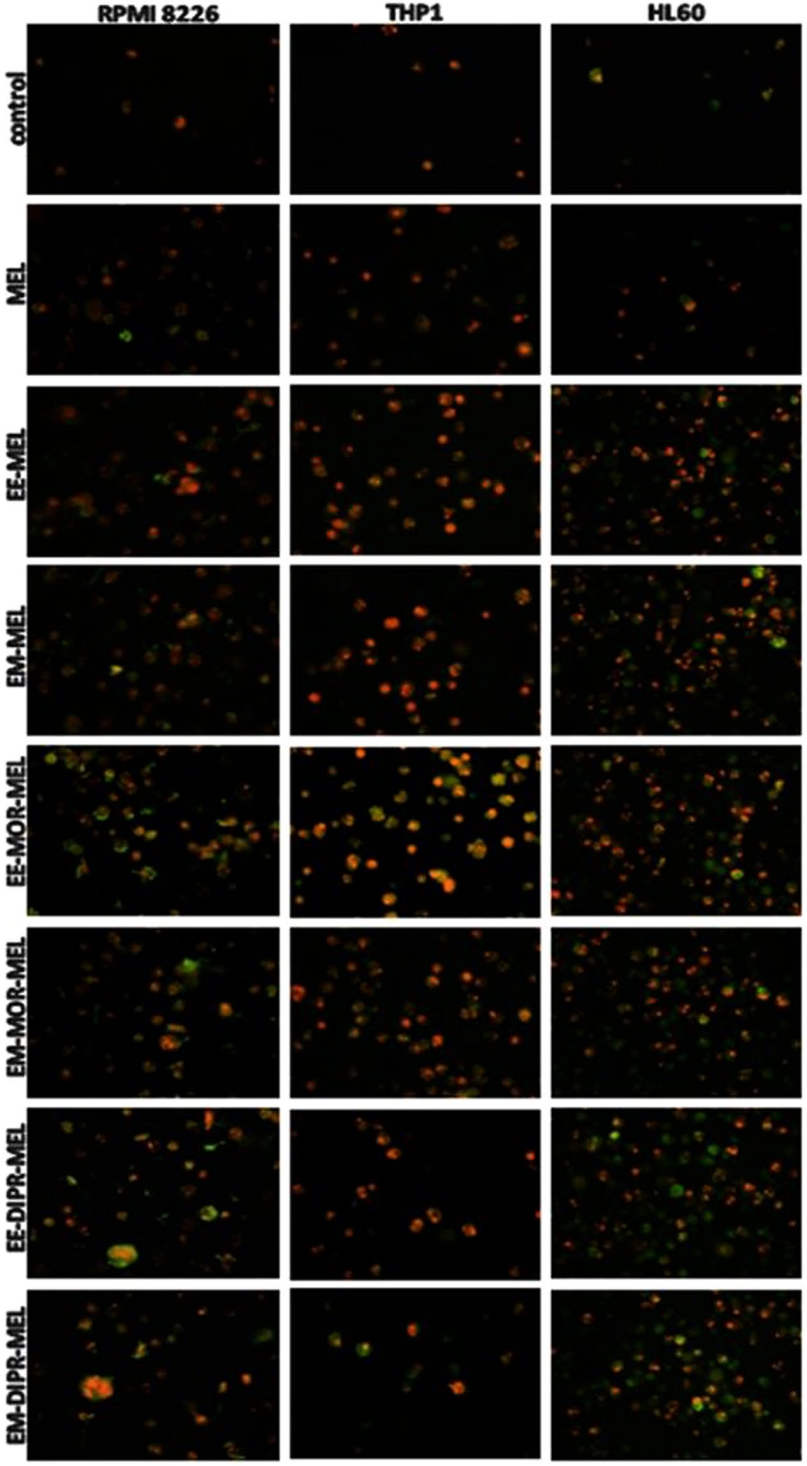
Fluorescence images of RPMI8226, HL60 and THP1 cells at 48 h after treatment with melphalan and its derivatives. The cells were stained with an annexin V/propidium iodide mixture and visualised by fluorescence microscopy, magnification ×200. After drug treatment, high green (derived from annexin V- fluorescein isothiocyanate, FITC) and red fluorescence (derived from propidium iodide) in cells with exposed phosphatidylserine (PS) indicated damage to the integrity of cellular membranes, characteristic symptoms of the late stages of programmed cell death. (For interpretation of the references to colours in this figure legend, the reader is referred to the web version of this article).

To evaluate the possible role of apoptotic features on the cytotoxicity of melphalan and its derivatives, the mode of cell death triggered by these compounds was investigated. Using annexin V/propidium iodide and Vybrant^®^ DyeCycle^™^ Violet/SYTOX^®^ AADvanced^™^ double staining methods, quantitative (Fig. 2) and qualitative (Fig. 3) assessments were made of molecular events connected with apoptosis. The average effect of unmodified and modified melphalan compounds on cell populations, as well as morphological changes at the single cell level was investigated.

As opposed to RPMI8226 and THP1 cells, HL60 cells were a little bit more susceptible to annexin V externalisation after exposure to the drugs. In all investigated cancer cells, following exposure to all derivatives, a pronounced increase in the number of late apoptotic cells was found after 48 h (approximately 80–90% for RPMI8226 and HL60 cells, and 20–50% for THP1), while a smaller increase (approximately 25% for RPMI8226, 35% for HL60 and 17% for THP1) was observed in cells treated with unmodified melphalan, for the same concentrations and time. It should also be taken into account that by 48 h of treatment, the percentage of the late apoptotic fraction for all investigated compounds increased greatly in comparison to the 24 h incubation time, whereas the early apoptotic fraction continued to strongly decrease. For the THP1 cell line at 48 h, EE-DIPR-MEL, EM-DIPR-MEL were the least potent derivatives at inducing apoptosis, with an effect comparable to that of the unmodified drug. Under the same conditions, a constant number of necrotic cells were observed for all cell lines. In the case of chromatin condensation measurements, similar results were observed, correlating with data from studies using annexin V/propidium iodide staining. In summary, biochemical changes connected with apoptotic cell death was dominant, whereas necrotic changes were not associated.

### Measurements of DNA damage by comet assay and the terminal deoxynucleotidyl transferase dUTP nick end labelling (TUNEL) method

**Figure 4:**
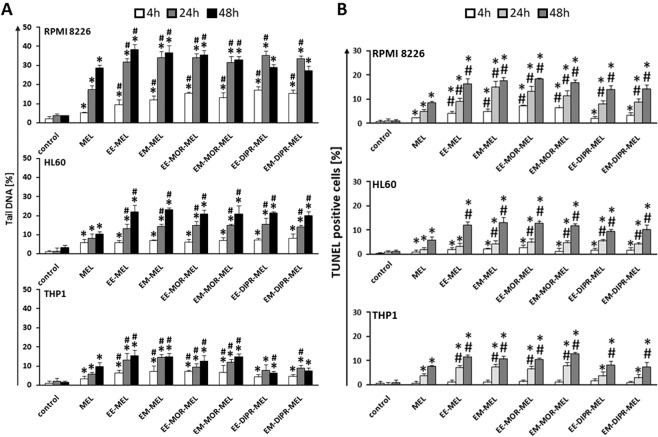
(**A**) Tail DNA (%) of cells treated with investigated compounds for 4, 24 and 48 h in RPMI8226, HL60 and THP1 cell lines. The number of analysed cells in each treatment was 100, and the analysis was repeated three times. (**B**) Influence of melphalan and its derivatives on the induction of DNA damage correlated with apoptosis in RPMI8226, HL60 and THP1 cancer cell lines, as estimated by TUNEL assay. Error bars denote SD.

In the current study the alkaline version of the DNA comet assay (pH > 13) was performed to assess the degree of DNA damage in RPMI8226, HL60 and THP1 cells. Quantitative data obtained from agarose gel electrophoresis are presented in Fig. 4A. Results indicated that THP1and HL60 cell lines were less susceptible to DNA damage by both melphalan and the melphalan derivatives, compared to RPMI8226 cells. Treatment with the modified drugs generated a higher level of DNA breaks in comparison to untreated (control) cells, as well as melphalan-treated cultures, especially after longer incubation times.

In myeloma cancer, after 48 h of treatment, the maximum level of cells with damaged DNA was observed for EE-MEL and EM-MEL (~38%). A slightly lower percentage of damaged cells was noted after treatment with EE-MOR-MEL and EM-MOR-MEL. EE-DIPR-MEL and EM-DIPR-MEL were the least potent compounds in inducing DNA damage, with an effect comparable to that of melphalan. A similar effect was observed in the THP1 cell line, where EE-DIPR-MEL and EM-DIPR-MEL caused DNA damage at the level of ~7% in comparison to EE-MEL and EM-MEL (~15%), and EE-MOR-MEL and EM-MOR-MEL (~13%). In HL60 cells, a 48 h incubation with modified drugs led to a similar increase in DNA in the comet tail (~21%). In order to confirm these results, an alternative method, TUNEL assay, was used to measure DNA damage (Fig. 4B).

All investigated cell lines showed an increase in the number of cells with damaged DNA after exposure to melphalan derivatives. Moreover, the sensitivity to DNA damage differed between these cell lines, and increased in the following order, THP1 < HL60 < RPMI8226, which correlated with susceptibility of cells to DNA damage as determined by comet assay. Once again, EE-DIPR-MEL and EM-DIPR-MEL derivatives were the least effective in comparison to EE-MEL, EM-MEL, EE-MOR-MEL and EM-MOR-MEL for the cell lines under evaluation.

### Detection of caspase-8, -9, -3/-7 activity

**Figure 5:**
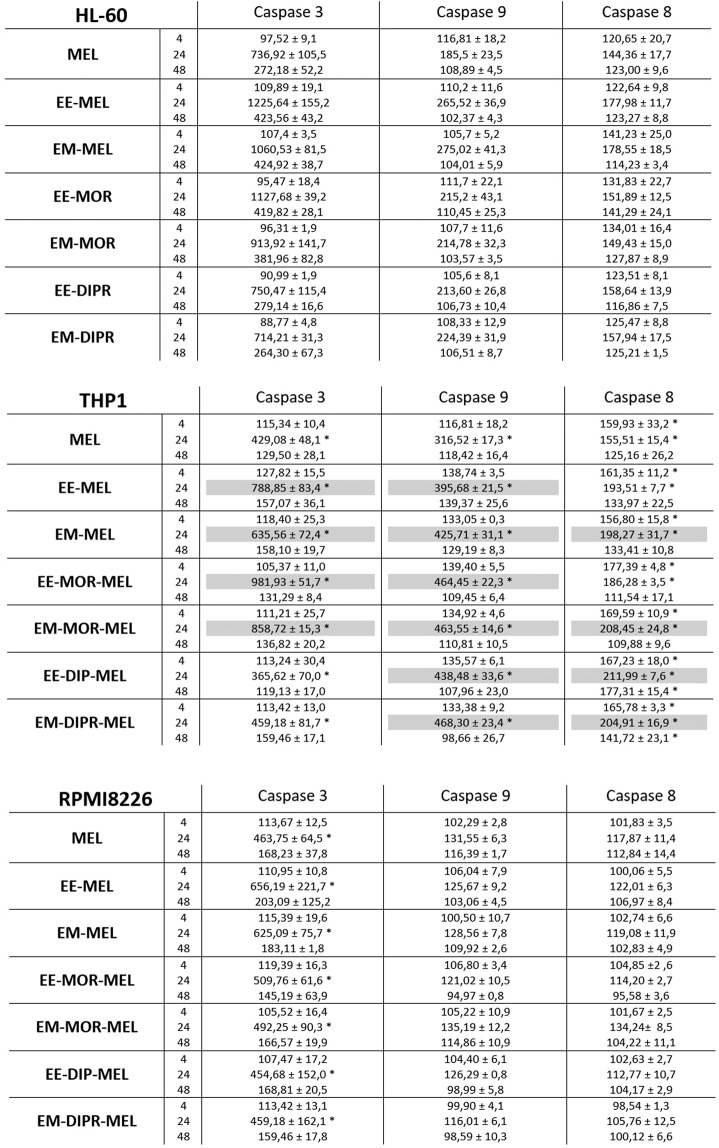
Changes in activity of caspase-9, caspase-8 and caspase-3 after exposure of RPMI8226, HL60, THP1 cell lines to melphalan and analogues for 4, 24, 48 h. The measurements were carried out in the presence or absence of inhibitors. The cells were treated with an IC_50_ dose of MEL and its derivatives. The final result obtained was a percentage activity of a particular caspase, where the fluorescence value (caspase-3) or luminescence (caspase-8 and -9) of the control, not treated with the compound, was taken as 100%. Each point represents the average ± SD of three independent experiments. Values shaded in grey indicate differences between samples incubated with new derivatives and those incubated with MEL.

Changes in caspase-3, -8 and -9 activity in HL60, THP1 and RPMI8226 cells exposed to melphalan derivatives and to MEL are shown in Fig. 5. The final results were expressed as percentage activity of a particular cysteine protease, where the fluorescence value (caspase-3/-7) or luminescence (caspase-8 and -9) of the control not treated with the compound was taken as 100%. The highest increase in caspase-3/-7 activity was observed after incubation for 24 h with all tested derivatives, EE-MEL, EM-MEL, EE-MOR-MEL, EM-MOR-MEL, EE-DIPR-MEL and EM-DIPR-MEL. The HL60 cell line was the most sensitive to activation of caspase-3/-7, followed by THP1 and then RPMI8226. In THP1 and HL60 cells, the highest increase in caspase-3/-7 activity was observed after incubation with the EE-MEL and EE-MOR-MEL derivatives. These values were 1.5 to 2-fold higher than for the reference drug (HL60, 736.92 ± 105.48%; THP1, 429.08 ± 48.10%), and were 1225.64 ± 155.22% (EE-MEL) and 1127.68 ± 39.24% (EE-MOR-MEL) for HL60, and 981.93 ± 51.79% (EE-MOR-MEL) and 788.85 ± 83.40% (EE-MEL) for THP-1. For RPMI8226 cells, increase in caspase -3/-7 activity were observed after 24 h incubation with EE-MEL (656.19 ± 221.7%) and EM-MEL (625.09 ± 75.7%) while the value for the reference drug was 463.75 ± 64.5% (Fig. 5).

In order to better understand the molecular mechanisms underlying the activation of caspase-3 in THP1, HL60 and RPMI8226 cells in response to the new derivatives, the measurement of caspase-8 and caspase-9 activity was examined. Significant changes were observed only in HL60 and THP1 cells. A strong increase in the activity of caspase-9 was noted after 24 hours of incubation with all the derivatives. The activity of this enzyme for MEL was 185.53 ± 23.12% (HL60) and 316.5 ± 17.31% (THP1). Results for all analogues were similar, and ranged from 213.60 ± 26.83% to 275.02 ± 41.32% for HL60, and from 395.68 ± 21.46% to 468.30 ± 23.43% for THP1. For THP1 cells, all tested compounds also caused an increase in caspase-8 activation after 4 and 24 hours of incubation, whereas in HL60 cells, changes were observed after 24 and 48 hours of incubation. In the case of RPMI8226 cells, no significant changes in the activation of this cysteine protease were observed after incubation with all compounds (MEL and MEL derivatives).

The experiments were also carried out simultaneously with caspase-3/-7, -8 and -9 activity inhibitors to ensure that the enzymes activated by the test compounds were the cysteine proteases being tested. Inhibitors caused a decrease in the percentage of the activity, indicating that the reactions were specific and resulted from the action of the tested compounds.

### Intracellular calcium measurement

**Figure 6:**
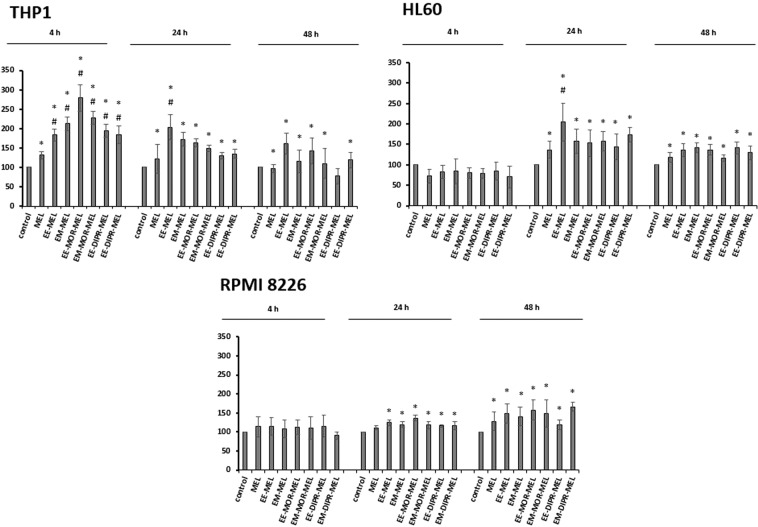
The effect of melphalan and its derivatives on Ca^2+^ concentration in THP1, HL60 and RPMI8226 cell lines. The cells were treated with an IC_50_ dose of the test compounds and then incubated for 2, 4, 24 or 48 h. The intensity of Fluo-4-NW probe fluorescence measured in control cells after 2, 24 or 48 h of incubation was taken as 100%. Each point represents the average ± SD of three independent experiments.

To examine whether the changes of intracellular calcium (Ca^2+^) were involved in apoptosis induced by melphalan analogues, the release of calcium was studied using the fluorescence probe Fluo-4-NW. Figure 6 shows that melphalan and its derivatives induced different effects on the distribution of calcium ions in HL60, THP1 and RPMI8226 cells. In treated cells, changes in calcium level were detected, which was strongly dependent on the cell line, the type of chemical modification of the basal compound and the duration of the treatment. Generally, all analogues caused greater changes in intracellular calcium level, compared to melphalan.

With regard to THP1 cells, the biggest changes were observed after short-term incubation with all tested compound (4 h). While the level of intracellular calcium for MEL was 131.27 ± 10.08%, results for all analogues were similar and ranged from 183.14 ± 22.86% to 279.15 ± 34.15%. Under these particular conditions, the greatest changes in the level of intracellular calcium ions were observed after treatment with EE-MOR-MEL (approx. 2.8-fold increase). Extending the incubation time of the experiment resulted in a gradual reduction in the percentage of calcium released with all the compounds.

By contrast, short-term incubation (4 h) of HL60 cells with the analogues did not cause any significant change in the distribution of calcium. However, the most striking changes (approx. 2-fold increase) were found after longer treatment (24 h) with the ethyl ester of melphalan (EE-MEL). Further, but lower increases (around 1.2–1.4 fold) were observed after longer incubation with all investigated compounds (48 h).

Surprisingly, an opposite effect was observed in RPMI 8226 cells. The maximal increase in the percentage of calcium level was noted at 48 h after treatment with melphalan analogues.

## Discussion

To strengthen the position of chemotherapy in fighting cancer more efficient methods of identifying new drugs need to be implemented. Identification of restrictions in chemical structure of certain groups of compounds in terms of their biological activity might constitute a valuable source of knowledge, useful in planning subsequent compound synthesis.

The object of this study was to identify melphalan derivatives with the enhanced cytotoxic activity in human cancer cells. The structure of melphalan molecule is notable for the presence of two modifiable functional groups, amino and carboxyl group. Systemic modification of both of these groups gives the possibility of extensive comparisons.

*In vitro* validation of cytotoxic, antiproliferative and proapoptotic properties of these compounds against various cancer cells, as well as results of investigation of their structure activity relationship (SAR) may provide a basis for the development of derivatives having optimal structure to serve as future anticancer drugs. For our research, RPMI8226- myeloma cancer, HL60- promyelocytic leukemia, and THP1- acute monocytic leukemia cell lines were chosen as haematological malignancy models.

This study used well known methods as screening tools. Initially, melphalan and its derivatives were evaluated for cytotoxicity in the selected model cells. Almost all derivatives, with the exception of MOR-MEL and DIPR-MEL, were recognized to be more toxic than the parent compound, MEL, in all three cell lines. Furthermore, significant differences in analogues’ toxicity against the cell lines were detected. The toxicity of derivatives was the highest against the HL60 and THP1 cell line, while RPMI8226 cells showed the lowest sensitivity. EM-MOR MEL and EE-MOR MEL showed the highest efficacy against cancer cell lines HL60 and THP1, while RPMI8226 cells were more sensitive to EE-MEL and EM-MEL. Pilot studies also showed that EE-MEL, EM-MEL, EM-MOR-MEL are less cytotoxic to normal peripheral blood mononuclear cells.

Considering the interaction of all the aforementioned compounds with the three cell lines, the most effective melphalan structure had a free amino group and a modified carboxyl group, which was either a methyl or ethyl ester. Esters are known to be useful in modification of the drug lipophilicity. Additionally, aliphatic esters generally enhance lipid solubility^19^. However it should be noted that the influence on modification activity in one part of a molecule is not easy to be determined unequivocally, even for one specific cell line, because it can depend, to a large extent, on modifications observed in other parts of the molecule. It should be taken into account that the anticancer effectiveness of drug is often combined with its dose and its accumulation in individual cells. Therefore various cell types could demonstrate different levels of sensitivity to identical doses of a drug. Comparison of the chemical modifications of the derivatives with their cytotoxicity results confirmed the importance of certain chemical groups. Hence, we shall be able to successfully plan the synthesis of melphalan derivatives with anticipated high cytotoxicity capacity.

Distinguishing between mechanisms that induce cancer cell death is extremely important in terms of drug efficacy. Therefore one of the main assumptions of our investigations was to obtain information about the mechanism of cell death induced by melphalan derivatives.

Inhibition and inability to undergo apoptosis is a critical point in the development of cancer and a major barrier to its effective treatment. Due to numerous genes’ mutation cancer cells gain immortality and are not annihilated by programmed cell death (PCD) and may proliferate excessively, which leads to tumor development and growth. Therefore the potential of chemotherapeutic agents and any cancer therapy to induce apoptosis of cancer cells is one of the most desirable properties. Given the above, principal aim of the study was to analyze the cytotoxicity of the tested melphalan derivatives and their contribution to cancer cell apoptosis.

Proposed detailed research assignments was aimed to estimate whether the melphalan derivatives can show proapoptotic activities in investigated cancer cells and if so, by which molecular mechanisms. For this reasons the effect of the investigated compounds on nucleic acid degradation (by comet and TUNEL assays) and parameters directly related to apoptosis (by PS externalisation and chromatin condensation) were measured. A number of characteristic biochemical changes occur at an early stages of apoptotic cell death. Unlike conventional cytotoxicity measurements which only assess parameters proportional to the degree of cell death, these parameters are measured for shorter incubation times. For this purpose, analysis of the contribution of apoptosis process in cytotoxicity of investigated compounds were examined after increasing times of incubation (4–48 h).

In this study DNA comet assay was performed under alkaline conditions. This method allowed detection of various types of DNA damages, such as single and double stranded DNA breaks (SSB and DSB respectively), DNA fragmentation induced by free radicals, cross-type DNA-DNA bonds and DNA-protein interactions^20^. This assay permits the quantification of DNA damage in a single cell preparation and is applicable to any eukaryotic cell. The assay can be used in both *in vitro* and *in vivo* testing and has been shown to be a powerful and sensitive predictor of genetic toxicity^21^. Our research clearly revealed that melphalan analogues generated a significant higher level of DNA breaks in comparison to unmodified drug, especially as a result of extended incubation times. Unexpectedly, in comparison to the comet assay, the TUNEL assay taken as an alternative method of DNA damage measurement connected with apoptosis, gave a poor signal response upon treatment with all test compounds. DNA is digested into regular oligonucleosomal fragments or multiples thereof. This phenomenon is the characteristic feature of apoptosis versus DNA degraded into irregular pieces in necrosis. However, in particular incidents DNA scission may stop and not progress into intranucleosomal fragments. As such, these apoptotic cells will be characterized by low levels of BrdU-FITC fluorescence. Hence, it is advised that other markers should be used in the identification of apoptotic cells than the ones dependent on the presence of DSB^22^. In addition, the DNA comet assay, used in this research, does not give an unambiguous answer to the question of whether DNA breaks are the result of mobilization of the apoptotic cell death machinery, because DNA may also be degraded in necrotic cells^20^. Criteria for apoptotic and necrotic death, including biochemical and molecular changes, indicate that both types of death are interdependent.

For these reasons, we studied cellular alterations connected with the leading processes responsible for cancer cell death activated by investigated derivatives, PS externalisation and chromatin condensation.

Morphological and biochemical changes such as the asymmetry of the cell membrane, nucleus shrink and compaction of chromatin are characteristic for the process of apoptosis. As a result of all complex molecular mechanisms involved in apoptotic process, progressive degradation of DNA occurs, which is dependent on active endonucleases. Thus, rating of apoptogenic properties of melphalan compounds were made on the basis of changes in plasma membrane (PS externalization) involved in apoptotic process.

Our studies have shown that all new analogues of melphalan induced apoptosis hallmarks; however, mainly at the late stages (irrespective of incubation time). More to the point, the results of the current study furnished proof that melphalan derivatives were considerably more effective against the three cell lines than the parent drug, MEL, and increased the anticancer activity.

Apoptosis, depending on the type of inducing agent, can take place with the participation of various evolutionarily conserved molecular mechanisms responsible for signal transmission initiating this process. Most proteolytic processes during apoptosis occur in the presence of a family of cysteine-dependent proteases catalysing the hydrolysis of proteins – caspases. Having confirmed that MEL and its derivatives induced apoptosis, the next stage of this study was aimed at determination of the apoptosis pathway (internal or external). For this purpose, we examined the activity of caspase-8, involved in the external pathway, caspase-9, which is a part of the internal apoptosis pathway, and caspase-3, which is involved in both pathways. In addition, the effect of the tested compounds on the increase of intracellular calcium levels was observed. HL60 was the most sensitive line for activation of caspase-3, followed by THP1 and RPMI8226, respectively. In case of THP1 and HL60, the highest increase in this cysteine protease was observed upon 24 hours of incubation with EE-MEL and EE-MOR-MEL. Contrary, for RPMI8226, the highest increase was found after incubation with methyl and ethyl melphalan esters.

The intrinsic apoptotic pathway is mediated by caspase-9 activation, which causes the release of mitochondrial cytochrome c and the formation of the apoptosome^23^. In THP1 and HL60 cells the strongest increase in caspase-9 activity was found after 24 h of incubation with the derivatives; changes that were statistically significant in comparison with cells incubated with MEL. In case of THP1 cells, this increase might be related to the high sensitivity of this line to oxidative stress and reactive oxygen species^24^. In HL60 and THP1, an increase in caspase-8 activity was also detected; however, the level of this cysteine protease was lower than for caspase-9. This suggested a dominance of the intrinsic apoptotic pathway.

No increase in caspase-9 or caspase-8 activity was observed for RPMI8226 cells. This might suggest the induction of cell death mechanisms other than apoptosis, such as mitotic catastrophe, autophagy, necroptosis or alternations of the apoptotic pathways. Pan *et al*. (2011) showed that melphalan, especially at low doses, induces autophagy in myeloma cells, and the use of inhibitors of this process significantly augments proapoptotic activity of DNA-damaging chemotherapy, both *in vitro* using MM cell lines or purified patient MM cells and *in vivo* in a human plasmacytoma xenograft mouse model^25^. It also should be mentioned that in addition to the best-known proteases - caspases involved in the effector phase of programmed cell death- there are other proteases like both cysteine (calpain, cathepsin B and L isoforms), aspartate (cathepsin D) and serine (granzyme B, AP24 protease). There have been suspicion that perhaps the proteolytic activity of calpain and caspase-3 is hierarchical. It has been observed that calpain activates (through proteolytic cleavage) caspase-3, -7 and -9. Activation of calpain by caspase-3 has also been reported. It is also possible that caspase-3 (but also -1 and -7) may be responsible for the proteolysis of calpastatin, thus causing the activation of calpain^26–29^.

The current studies indicated that the cellular response to the test compounds varied depending on the type of cells (leukemia and myeloma cells). Therefore, further research will be carried out to describe the type of cell death caused by derivatives in a detailed way, including the possibility of the simultaneous participation of different pathways leading to cell death.

It was demonstrated that changes in calcium homeostasis play a key role in necrotic and apoptotic processes. Moreover, additional types of cell death, particularly anoikis as well as autophagy, are modulated by transient Ca^2+^ levels^27^. Ca^2+^ ions are also indispensable for the activation of some enzymes that reorganize the chromatin complex and its availability on the nucleolytic attack and also affect, directly or indirectly, the induction of expression (initiation of transcriptional activity) of genes associated with the execution of the PCD^26–31^.

All the analogues investigated in the current study caused greater changes in intracellular calcium levels. In THP-1 cells, the biggest changes were observed after short-term incubation (4 h), mainly with EM-MOR-MEL. In case of HL60 cells, significant changes were observed after incubation for 24 hours only, mainly with the ethyl ester of melphalan (EE-MEL). However, in RPMI8226 cells, maximal increase in the percentage of calcium level was noted upon treatment for 48 h with all melphalan analogues. These results were consistent with the previously reported studies on the induction of apoptosis in HL60, THP1 and RPMI8226 cells.

## Conclusions

A new series of nine melphalan analogues were designed, synthesised and tested. A comprehensive approach to the structure-antiproliferative activity relationship was proposed by attempting to compare the modification of both the carboxyl and amino groups of the parent molecule. As a result of that comparison, novel analogues EM-MOR-MEL and EE-MOR-MEL showed better activities (cytotoxicity, genotoxicity and ability to induce apoptosis) than melphalan, a drug still on the market.

**Figure 7:**
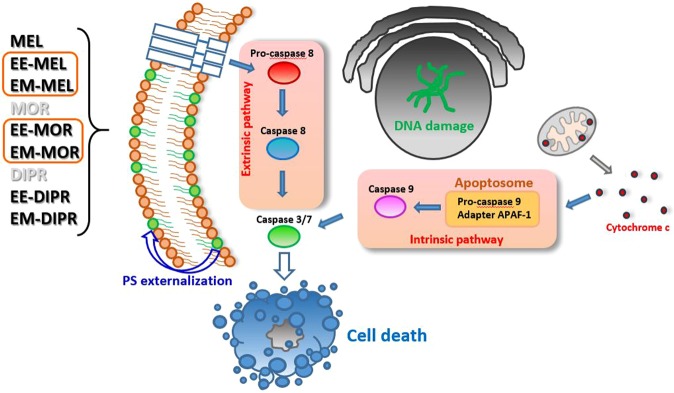
Proposed model of the molecular and cellular responses to the new MEL derivatives.

All investigated derivatives (with a particular mention for EE-MEL, EM-MEL, EE-MOR-MEL and EM-MOR-MEL) caused significant changes in cells, compared to those treated with melphalan, indicating once again that these proposed modifications might serve as a potent drug therapeutic system. However, the presented study is limited to only *in vitro* cell line data. The results generate hypothesis and require validation with *in vivo* experiments. These studies allowed us to select the most active compounds for further, more detailed investigations, significantly advancing our understanding of toxic mechanisms of specific chemicals (Fig. 7).

## Materials and methods

### Chemicals

**Figure 8:**
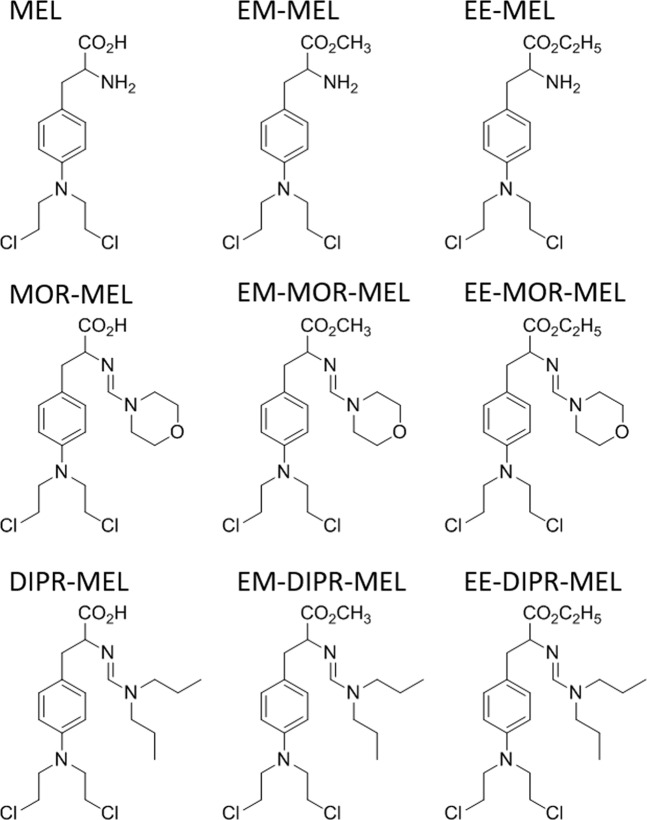
Chemical structures of melphalan derivatives.

All tested compounds (Fig. 8) with purity over 98.5% (HPLC) were synthesized in the ŁUKASIEWICZ Research Network-Institute of Biotechnology and Antibiotics, Warsaw, Poland, method described in the Polish Patent PL220880 B1 (Espacenet database). HPLC analysis were performed using a Waters liquid chromatographic system consisting of DAD detector. A Chromolith Performance RP-18e (100-4.6 mm) column was used. at constant flow rate of 1.0 ml/min. The mobile phase consisted of laurylosulfate buffer and acetonitrile; solvent were filtered under vacuum, mixed and degassed by a helium before use.

### Cell culture

RPMI8226 (ATCC^®^ CCL-155^™^) myeloma cancer cells, HL60 (ATCC^®^ CCL-240^™^) promyelocytic leukemia cells and THP1 (ATCC^®^ TIB-202^™^) acute monocytic leukemia cells were purchased from American Type Culture Collection (ATCC, Rockville, USA). Peripheral Blood Mononuclear Cells (PBMC) were isolated from buffy coat obtained from the Central Blood Bank (Lodz, Poland). PBMCs were isolated with Histopaque 1077 (Sigma Aldrich) at density gradient by centrifugation at 300 × g for 15 min.

All investigated cells were grown as a monolayer with RPMI 1640 medium, 1% phytohemaglutinin (only in PBMCs growth medium) supplemented with 10% fetal bovine serum, penicillin (10 U/ml) and streptomycin (50 μg/ml), in standard conditions: 37 °C, 100% humidity, the atmosphere being 5% CO_2_ and 95% air. The cell viability was systematically controlled using trypan blue (0.4%, Sigma). In all experiments, cells in logarithmic phase of growth were used when their viability was above 95%.

### Cytotoxicity assay

The effect of melphalan and its derivatives on RPMI8226, THP1, HL60 and PBMCs growth was determined by using resazurin viability assay. Cells subcultured into 96-well black plates at a density of 1.5 × 10^4^ cells/well were incubated with various concentrations of selected compounds at 37 °C for 48 h. After incubation resazurin solution was added to each well (10 µg/ml, final concentration) and the plates were incubated for 90 minutes. Fluorescence measurement was performed at ~530 nm excitation and ~590 nm emission using an Fluoroskan Ascent FL plate reader (Labsystems, Sweden).

### Measurements of phosphatidylserine externalization

Double staining of cells with Annexin V and propidium iodide (PI) was used according to the protocol described in our previous article^32^. This method is a useful tool for distinguishing viable cells (unstained with either fluorochrome) from early apoptotic cells (stained only with Annexin V), late apoptotic (stained with Annexin V and propidium iodide) and necrotic (dead) cells (stained only with PI).

Briefly, 5 × 10^5^ control and drug treated cells were washed with cold PBS and resuspended in 500 μl binding buffer (delivered from producer) that contained 5 μl of Annexin V fluorescein isothiocyanate (FITC), 5 μl of PI and stained for 15 minutes in room temperature. Finally, at least 10^4^ cells were analyzed for FITC and PI fluorescence (Ex ~488 nm; Em ~530 nm) using a flow cytometer (LSR II, Becton Dickinson). The cells stained with the annexin V/propidium iodide mixture were then visualized using fluorescence microscopy (Olympus IX70, Japan), magnification × 200.

### Measurements of chromatin condensation

Violet Chromatin Condensation/Dead Cell Apoptosis Kit with Vybrant® DyeCycle™ Violet and SYTOX® AADvanced™ was used in order to examine chromatin condensation during apoptosis (Molecular probes^®^, Invitrogen^™^). Briefly, 5 × 10^5^ control and drug treated cells were washed and suspended in 1 ml of Hank’s Balanced Salt Solution buffer (HBSS) containing Vybrant^®^ DyeCycle^™^ Violet and SYTOX^®^ AADvanced™ dyes, according to manufacturer’s protocol. Immediately after the incubation period (30 min, protected from light), stained cells were analyzed without washing by flow cytometry (LSR II, Becton Dickinson), using ~405/488 nm dual excitation while measuring the fluorescence emission at ~440/660 nm.

### Measurements of DNA damage – comet assay

Comet assay was performed under alkaline conditions according to the protocol described in our previous article^33^. Cells were suspended in 0.75% low melting point agarose in PBS, pH 7.4. Next, 50 μl of this suspension was spread on frosted microscope slides precoated with 1% normal melting agarose. After gelling, the slides were treated with lysing buffer consisting of 2.5 M NaCl, 100 mM EDTA, 1% Triton X-100, 10% DMSO and 10 mM Tris, pH 10 at 4 °C for 1 h. Then slides were placed in the electrophoresis solution (300 mM NaOH, 1 mM EDTA, pH > 13) for 40 min to allow DNA unwinding. Electrophoresis was carried out at 0.73 V/cm and 30 mA for 30 min. Then the slides were stained with 2 μg/ml DAPI. All these steps were performed in the dark to prevent additional DNA damage. 100 randomly selected cells from each slide were analyzed using fluorescence microscope (Olympus IX70, Tokyo, Japan) equipped with a CC12 video camera, a UV filter and image analysis system - CaspLab v. 1.2.3b2. The percentage of DNA in the comet tail was chosen as an indicator of DNA damage.

### Measurements of DNA damage during apoptosis – TUNEL assay

ApoBrdU DNA Fragmentation Assay Kit (BioVision) was used in order to examine DNA damage during apoptosis according to the protocol described in our previous article^34^. This method enables the detection of the early stage of apoptosis by labeling 3′OH ends of single- and double-stranded DNA fragments with Br-dUTP (bromolated deoxyuridine triphosphate nucleotides). The Br-dUTP fragments are detected by the fluorescein labeled anti-BrdU monoclonal antibody, which enables a brighter signal. Control and drug treated cells were fixed in a 4% paraformaldehyde freshly prepared in PBS and incubated for 1 h at 37 °C in DNA Labeling Solution containing a TdT Reaction Buffer, a terminal deoxynucleotidyl transferase (TdT) and the Br-dUTP. Next, the cells were resuspended in an antibody solution containing an anti-BrdU-FITC antibody (in total darkness for 30 min at room temperature) and incubated with the propidium iodide/RNase A solution. The cells fluorescence was measured with the flow-cytometry (LSR II, Becton Dickinson). The green fluorescence of FITC at 520 nm and the red fluorescence of propidium iodide at 623 nm were detected. The number of TUNEL-positive cells was expressed as a percentage of the total number of cells in the sample.

### Measurement of caspase 3/7, 8 and 9 activation

The activity of caspases-3 and -7 were estimated with Apo-ONE^®^ Homogeneous Caspase 3/7 Assay and Caspase-Glo^®^ 9/8 Assay Systems according to the manufacturer’s protocols (Promega Corporation, Madison, WI, USA). Measurement of caspases activation in control and treated cells seeded in 96-well plates (black or white), was recorded by monitoring changes in the fluorescence (caspase-3/7) or luminescence (caspase-8 and -9) after 4 h, 24 h and 48 h incubation of cells with investigated compounds. The intensity of fluorescence or luminescence were measured using a Fluoroskan Ascent FL plate reader (Labsystem, Sweden). Cysteine proteases activity were expressed as a ratio of fluorescence or luminescence of the treated samples relative to the corresponding untreated controls taken as 100%. The proper inhibitors were used in the control experiments to confirm that the observed fluorescence in both the control and the drug-treated cells is due to caspase-3, -9, -8 presences in the samples^32^.

### Intracellular calcium measurement

Intracellular calcium level was determined using the fluorescent probe Fluo-4NW according to the protocol described in our previous article^32^. After entering the cell, Fluo-4NW is converted by cytosolic hydrolases to the active form, having the possibility of binding of calcium ions. As a result of joining of calcium ions, the probe emits fluorescence (λ_em_ = 538 nm) after excitation with light of wavelength 485 nm.

Cells were cultured and treated with investigated compounds on Petri dishes. Next, the cells were plated in 96-well black fluorimetric plates (2 × 10^4^ cells/well). The growth medium was removed and the cells were washed with PBS in order to eliminate sources of baseline fluorescence. Finally a dye loading solution (Fluo-4-NW dye, 4-[(Dipropylamino)sulfonyl] benzoic acid (Probenecid) - used to inhibit extrusion of the indicator out of the cell by organic anion transporters, Hanks’ balanced salt solution (HBSS), 20 mM N-(2- hydroxyethyl)piperazine-N’-(2-ethanesulfonic acid) buffer solution (HEPES)) was added in a volume of 100 μl per well and incubated for 30 min in the total darkness at 37 °C, and then for next 30 min. at room temperature. The measurement was done on Fluoroskan Ascent FL microplate reader (Labsystems, Sweden) using 494 nm excitation and 516 nm emission wavelengths.

### Statistical analysis

The data was presented as a mean ± S.D. An analysis of ANOVA variance with a Tukey post hoc test was used for multiple comparisons. All statistics were calculated using the STATISTICA program (StatSoft, Tulsa, OK, USA). A p-value of < 0.05 was considered significant. All figures contain identical descriptions for statistically significant changes: *p < 0.05 statistically significant differences in comparison to control cells, ^#^p < 0.05 statistically significant differences between samples incubated with melphalan and melphalan derivatives.
